# The effect of ethanol on the habit and *in vitro* aerodynamic results of dry powder inhalation formulations containing ciprofloxacin hydrochloride

**DOI:** 10.1016/j.ajps.2021.04.003

**Published:** 2021-05-28

**Authors:** Edit Benke, Christina Winter, Piroska Szabó-Révész, Eva Roblegg, Rita Ambrus

**Affiliations:** a Institute of Pharmaceutical Technology and Regulatory Affairs, University of Szeged, Szeged H-6720, Hungary; b Institute of Pharmaceutical Sciences, Pharmaceutical Technology and Biopharmacy, University of Graz, Universitätsplatz 1, Graz A-8010, Austria; c Research Center Pharmaceutical Engineering GmbH, Inffeldgasse 13, Graz A-8010, Austria

**Keywords:** Dry powder inhaler, Ethanol, Particle engineering, Roughness, Aerodynamic properties

## Abstract

In the case of dry powder inhalation systems (DPIs), the development of carrier-free formulations has gained increased attention. Thereby, spray-drying is a promising technology and is widely used to produce carrier-free DPIs. Numerous works have been published about the co-spray-drying of active ingredients with various solid excipients and their effect on the physicochemical characteristics and aerodynamic properties of the formulations. However, only a few studies have been reported about the role of the solvents used in the stock solutions of spray-dried formulations. In the present work, DPI microcomposites containing ciprofloxacin hydrochloride were prepared by spray-drying in the presence of different ethanol concentrations. The work expresses the roughness, depth and width of the dimples for particle size as a novel calculation possibility, and as a correlation between the MMAD/D_0.5_ ratio and correlating it with cohesion work, these new terms and correlations have not been published – to the best of our knowledge – which has resulted in gap-filling findings. As a result, different proportions of solvent mixtures could be interpreted and placed in a new perspective, in which the influence of different concentrations of ethanol on the habit of the DPI formulations, and thus on *in vitro* aerodynamic results. Based on these, it became clear why we obtained the best *in vitro* aerodynamic results for DPI formulation containing 30% ethanol in the stock solution.

## Graphical abstract

Dry powder inhalation (DPI) formulations containing ciprofloxacin hydrochloride (CIP) as a model drug were prepared by spray-drying in the presence of different ethanol concentrations. The role of ethanol on the habit and aerodynamics of the DPI samples was examined in detail. The roughness, depth and width of the dimples were uniquely expressed. A new correlation was determined between the mass median aerodynamic diameter (MMAD)/D (0.5) ratio and cohesion work (Wc). DPI formulation containing 30% ethanol in the stock solution had the best *in vitro* aerodynamic results.

**Figure:**
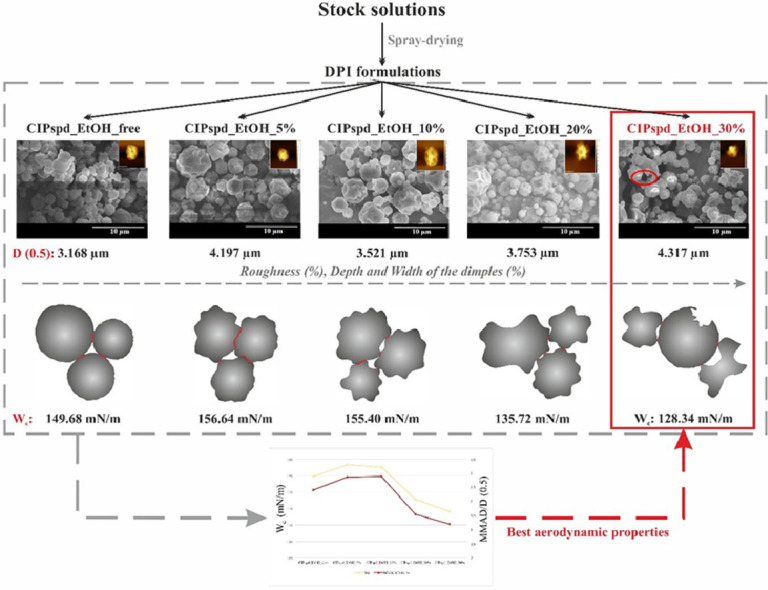

## 1 Introduction

Drug administration through the pulmonary route allows the treatment of local and systemic diseases [1]. Compared to oral drug delivery, there are several arguments in favor of drug delivery through the lungs as it is a non-invasive procedure [2]. Moreover, the active pharmaceutical ingredient (API) can reach the C_max_ value within minutes [3] and a much lower average dose of one-tenth of the oral dose is sufficient to achieve the same therapeutic effect [4]. These can be explained by the fact that the gastrointestinal (GI) tract is circumvented, thus, hepatic first-pass metabolism and/or enzymatic inactivation along the GI tract do not take place [5,6]. Furthermore, the side effect profile of the applied drug may be more favorable compared to oral administration [7]. Due to the aforementioned beneficial properties of pulmonary drug delivery, a large number of APIs have recently been tested [8,9].

With regard to pulmonary drug administration, the development of dry powder inhaler (DPI) systems has recently become more focused on internationally compared to pressured metered-dose inhalers and nebulizers [10]. In the case of DPIs, carrier-based formulations are substantially present on the market (on average, they still have 20%−40% fine particle fraction results) [11,12]. There has been a greater emphasis on the fabrication of carrier-free DPIs in recent years. In this respect, the use of excipients and the application of advanced technological solutions in the production of formulations (*e.g.*, spray-drying, spray-freeze-drying, and supercritical-fluid technology) make it possible to achieve favorable aerodynamic properties, leaving the large carrier particles in the formulation [13]. Spray-drying proves to be the most favorable manufacturing technique for carrier-free DPIs in terms of the advantages and the disadvantages of the above-mentioned technological solutions. It can be said that this method is a stepwise, relatively fast and simple, cost-effective, automated, reproducible technology, which can affect the morphology, size, and density of the samples by changing the applied preparation parameters. Disadvantages include a relatively high temperature during sample preparation and amorphization [14,15]. The former should not be a problem if the APIs and excipients are carefully selected [16], [17], [18]. The amorphous form is particularly advantageous for DPIs in terms of aerodynamics [19].

The role of a number of solid excipients in the co-spray-drying of DPIs has been investigated. Positive findings with several types of polymers have also been reported by the preparation of non-porous and porous carrier-free DPI formulations. Using PVA (polyvinyl alcohol) and PVP (polyvinylpyrrolidone), these excipients coat the surface of the particles, thereby helping to reduce interparticle interactions, and thus prevent agglomeration [20]. PLGA (poly-lactic-co-glycolic acid) is mainly applied as a matrix carrier [21,22]. Amino acids are also widely used. For example, by the production of DPIs, it has been confirmed that leucine undergoes structural changes during spray-drying, coating the drug particles, thereby providing protection against moisture, reducing interparticle interactions, and increasing the dispersity of the samples [23], [24], [25]. Moreover, the study of different analogs (D and L-leucine, Trileucine and Isoleucine) could be also found in the literature. In addition, experiments have been performed with, for example, glycine and alanine [26], [27], [28]. Another larger group of excipients that could be applied by co-spray-drying are lipids. In this case, improvements have been reported by matrix-forming or coating properties, and the role of the absorption enhancer has already been confirmed. As regards other excipients, mention may be made, about sodium stearate, which has a moisture protection role [29], or ammonium carbonate/bicarbonate, which has a pore-forming and density-reducing effect [30], [31], [32].

However, during spray-drying, it is important to study the role of solvents in addition to the solid excipients applied. Nevertheless, very few works have been published on this topic. Specifically, only the role of ethanol in spray-dried DPI formulations has been studied by Belotti et al. [33], Gilani et al. [34] and Ji et al. [35]. In each case, a remarkable difference in micrometric properties was found. Belotti et al. applied 0–10% of ethanol concentration and varying spray-drying preparation parameters using Central Composite Design, and the results were also explained by the change in the Peclet number. Gilani et al. used a 50%−100% of ethanol concentration. Ji et al. performed experiments on lysozyme using 0–80% of ethanol in the stock solution, but the stability of these formulations was also investigated. Several groups such as Rabbani and Seville [36] and Boraey et al. [37] varied the concentration of ethanol and used a solid excipient such as leucine in various concentrations, but these publications do not only detail the role of ethanol. The above-mentioned articles regarded ethanol as an excipient influencing the physicochemical properties of the particles/various parameters of the microcomposites rather than as a classical co-solvent [33,34,37]. Furthermore, in several publications, the use of organic solvent for DPI formulations in the spray-drying stock solution can be found, but without an explanation of the applied concentration [38], [39], [40].

The present research aims to highlight that how different proportions of solvent mixtures (distilled water, ethanol), especially ethanol concentrations, used in the spray-dried preparation of DPI samples containing ciprofloxacin hydrochloride affect the habit of these powders, thus the *in vitro* aerodynamic results. This work seeks to put into new perspective the experience published, using new calculated terms and contexts, to give new explanations and build up the sound basic knowledge on this subject.

## 2 Materials and methods

### 2.1 Materials

Micronized ciprofloxacin hydrochloride (CIP) (D50: 5.09 µm) as a fluoroquinolone antibiotic drug was kindly supplied by Teva Pharmaceutical Works Ltd. (Debrecen, Hungary). 96% ethanol (EtOH) was obtained from AppliChem GmbH (Darmstadt, Germany). Both raw materials used were of pharmacopoeial quality.

### 2.2 Methods

#### 2.2.1 Preparation of the DPI formulations

DPI samples were prepared from aqueous solutions of CIP. The stock solution of one sample did not contain EtOH, in the other cases, EtOH was used as a liquid excipient at different concentrations (5%, 10%, 20% and 30%, Table 1), and the applied temperature was 65 °C. The solubility of CIP was tested in the applied solvent mixtures before preparing the samples. The solubility values of CIP were 0.094 g/ml in distilled water and 0.072 g/ml in a 70:30 mixture of distilled water and EtOH at 65 °C with stirring for 2 h at 200 rpm. The CIP concentration in the stock solutions was 0.015 g/ml.

**Table 1:** Composition of the stock solution – used for spray-drying – of the samples and the yield of these processes.

Formulations	CIP (g)	EtOH (g)	Distilled water (g)	Spray-drying yield (%)
CIPspd_EtOH_free	3.00	–	ad 200.0	71.3
CIPspd_EtOH_5%	3.00	10.0	ad 200.0	78.3
CIPspd_EtOH_10%	3.00	20.0	ad 200.0	75.7
CIPspd_EtOH_20%	3.00	40.0	ad 200.0	76.3
CIPspd_EtOH_30%	3.00	60.0	ad 200.0	77.1

The spray-drying process was implemented with the Büchi B-191 apparatus (Mini Spray Dryer, Büchi, Switzerland) and the following parameters were applied: the inlet heating temperature was 130 °C, the outlet temperature was approximately 80 °C, the drying air flow rate was 75%, the sample pump speed was 5%, and the compressed air flow rate was 600 l/h [41,42]. The yield after the spray-drying was between 75% and 80% for each DPI formulation, which can be said to be appropriate by this technique [35,37,43]. The samples were named based on the ethanol concentrations used in the stock solutions.

#### 2.2.2 Structural analysis

The structure of the samples was examined with the application of the BRUKER D8 Advance X-ray powder diffractometer (XRPD, Bruker AXS GmbH, Karlsruhe, Germany). Cu K λI radiation (*λ* = 1.5406 Å) was the radiation source. The investigated formulations were scanned at 40 kV and 40 mA, the angular range was 3–40° 2θ, at a step time of 0.1 s/step and a step size of 0.010° For the evaluation, the DIFFRACT plus EVA software (Bruker, Brussels, Belgium) was used. The XRPD diffractograms were corrected by Kα2, smoothed and evaluated after background removal.

#### 2.2.3 Scanning electron microscopy (SEM)

To examine the morphology of the samples, SEM was applied (Hitachi S4700, Hitachi Scientific Ltd., Tokyo, Japan) at 10 kV. The raw CIP and the DPI formulations were coated with the help of a sputter coater (Bio-Rad SC 502, VG Microtech, Uckfield, UK) – with gold-palladium (90 s), under an argon atmosphere in a high vacuum evaporator – to induce electrical conductivity on the surface of the samples. The used air pressure was 1.3–13.0 MPa.

#### 2.2.4 Atomic force microscopy (AFM) and the expressed values

Prior to AFM-Imaging, the particles were carefully scattered on a double-sided adhesive tape for fixation during the measurement. AFM-Imaging was carried out with a FLexAFM atomic force microscope equipped with C3000 control software (Nanosurf AG, Liestal, Switzerland). Non-coated TAP-300-Al-G cantilevers (BudgetSensors, Sofia, Bulgaria) with a nominal resonant frequency of 300 kHz were used in phase contrast mode as an extension of the dynamic force mode. The data were processed and evaluated using the Gwyddion 2.55 software (Czech Metrology Institute, Brno, Czech Republic).

The calculation of the root means square roughness (R_RMS_) was performed based on Eq. 1. by applying Gwyddion software. For each formulation, a minimum of five [44] particles and 0.5 µm × 0.5 µm areas per particle were examined at a minimum of 3 different locations.

(1) $$R_{RMS}=\sqrt{\frac{1}{n}\sum_{i=1}^{n}y_{i}^{2}}$$

Thereby, *n* is the number of data points in a topographical profile and *y_i_* is the distance of asperities (i) from the center line [44].

For a given particle, knowing the average R_RMS_ (R_RMS (average)_) and the average diameter (d), which is the mean of 10 diameters determined in different directions, the roughness value for the particle size was expressed according to the following equation:

(2) $$\text{Roughness}\%=\frac{R_{RMS\left(average\right)}}{d}\times 100$$

Furthermore, with the help of the above-mentioned software, it was possible to measure only individual depressions/dimples of the particle surface. For each particle, at least three dimples were taken as a basis for expressing the average values (depth and width of the dimples_(average)_). Knowing these values and also the value of d of the given particle mentioned above, we determined the values of depth and width of the dimples for the particle size (Eq. 3 and 4). For each sample, a calculation was performed for a minimum of five particles.

(3) $$\text{Depth}\;\text{of}\;\text{the}\;\text{dimples}\%\frac{Depth\;of\;the\;dimples_{\left(average\right)}}{d}\times 100$$

(4) $$\text{Width}\;\text{of}\;\text{the}\;\text{dimples}\%\frac{Width\;of\;the\;dimples_{\left(average\right)}}{d}\times 100$$

#### 2.2.5 Particle size analysis

The determination of the D_0.1_, D_0.5_, and D_0.9_ values was carried out with laser diffraction (Malvern Mastersizer Scirocco 2000; Malvern Instruments Ltd., Worcestershire, UK). Approximately 0.5 g of the samples were filled into the feeder tray. The following settings/parameters were applied: dry analysis method, official refractive index of CIP from the Malvern database, 2.0 bars dispersion air pressure, 75% vibration feed, 12 s measuring time. For each sample, three parallel investigations were executed. The span value was calculated based on the following equation [45]:

(5) $$\text{Span}=\frac{D_{0.9}-D_{0.1}}{D_{0.5}}$$

#### 2.2.6 Determination of the bulk and tapped density, Carr index and Hausner ratio

For the determination of the densities, the samples were filled in the 5 ml tared graduated cylinders and the bulk density (ρ_bulk_) values came from the mass/volume ratios. In the cases of tapped density (ρ_tap_), the STAV 2003 Stampfvolumeter (Engelsmann A.G., Luwigshafen, Germany) was applied as tapping equipment (tapped 1250 times [46]) and the results were the mass/tapped volume ratios. All formulations were investigated in triplicate. Furthermore, Carr index and the Hausner ratio were determined with the equations below [47]:

(6) $$\text{Carr}\;\text{index}=\left[\frac{\rho_{tap}-\rho_{bulk}}{\rho_{tap}}\right]\times 100$$

(7) $$\text{Hausner}\;\text{ratio}=\frac{\rho_{tap}}{\rho_{bulk}}$$

#### 2.2.7 Measurement of the residual water content

The residual water content of the samples was analytically determined with Karl Fischer (KF) volumetric titration. The investigations were carried out using the TitroLine KF (SI Analytics, Mainz, Germany) titrator. The DPI sample (130 mg) was dissolved in water-free methanol prior to the analysis. The dissolved sample was injected into the reaction cell and titrated with 4.01 mg/ml of Apura® CombiTitrant 5 one-component reagent for volumetric Karl Fischer titration (Merck KGaA, Darmstadt, Germany). As a standard, HYDRANAL KF reagent (Honeywell Fluka™ Water Standard Oven 220–230 °C, Fisher Scientific, Germany) was used. The residual water content of the formulations was then accessed via endpoint titration. All samples were measured in triplicate.

#### 2.2.8 Measurement of the residual EtOH content

The residual EtOH content of the formulations was measured by the Mettler Toledo TG 821e thermal analysis system (TG) connected with a quadrupole mass spectrometer (MS, Pfeiffer Vacuum, model ThermostarTM GSD 320) and analyzed with the STARe thermal analysis program V9.1 (Mettler Inc., Schwerzenbach, Switzerland). In carrying out the measurement, 3–5 mg per formulation was weighed into 40 µ l aluminum pans. The start temperature was 25 °C, the end temperature was 350 °C, the used heating rate was 10 °C/min. The investigation was made in a nitrogen atmosphere (constant nitrogen gas flow: 10 ml/min). Contact between the TG and the MS was performed with a silica capillary maintained at 120 °C.

#### 2.2.9 Determination of cohesion work

The determination of cohesion work (W_c_) was carried out by applying a Dataphysics OCA 20 apparatus (Dataphysics Inc. GmbH, Germany). In the case of each sample, pastilles were pressed (0.10 g powder and 1 ton compression force) with the hydraulic press (Perkin Elmer, Waltham, USA). For each sample, three pastilles were dropped with distilled water as a polar liquid, and three pastilles were dropped with diiodomethane as a dispersion liquid. At each drop, the change of the contact angle (Θ) in a time interval of 1–25 s was measured by the above-mentioned apparatus and in each case, we applied to the following calculation at the same second determining contact angle values. Surface free energy (γ_s_) consists of a polar ($\gamma_{s}^{d}$) and a dispersed part ($\gamma_{s}^{p}$), therefore, $\left(\gamma_{s}=\gamma_{s}^{d}+\gamma_{s}^{p}\right)$. It was calculated from the Wu-equation [48]:

(8) $$\left(1+\cos\Theta \right)\gamma_{1}=\frac{4\left(\gamma_{s}^{d}\gamma_{1}^{d}\right)}{\gamma_{s}^{d}+\gamma_{1}^{d}}+\frac{4\left(\gamma_{s}^{p}\gamma_{1}^{p}\right)}{\gamma_{s}^{p}+\gamma_{1}^{p}}$$

where Θ = contact angle; γ = surface free energy; *s* = solid phase; *l* = liquid phase; *d* = dispersion component; *p* = polar component

Since the surface tension of the applied liquids is known in the literature $\left(\gamma_{1}=\gamma_{1}^{d}+\gamma_{1}^{p}\right)$: distilled water γ^p^= 50.2 mN/m, γ^d^ = 22.6 mN/m and diiodomethane γ^p^ = 1.8 mN/m, γ^d^ = 49 mN/m [49]. Only the disperse ($\gamma_{s}^{d}$) and the polar component ($\gamma_{s}^{p}$) are unknowns in this equation, these can be expressed [48]. Thus, surface free energy (γ_s_) can be determined, the double of which is W_c_ [50]:

(9) $$W_{c}=2\times \gamma_{s}$$

#### 2.2.10 In vitro test with the Andersen Cascade Impactor

**Fig. 1:**
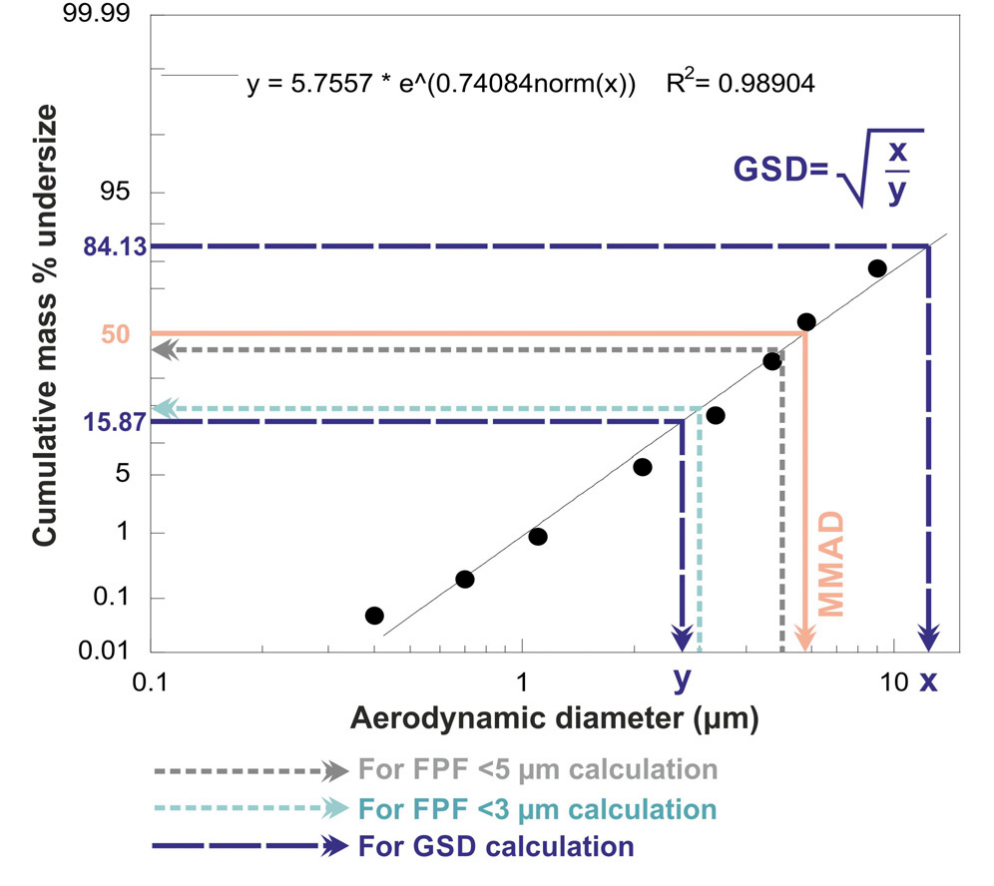
The process for determining the aerodynamic properties (with the help of the KaleidaGraph 4.0 software).

The *in vitro* inhalation test was implemented with the Andersen Cascade Impactor (ACI) (Copley Scientific Ltd., Nottingham, UK). This equipment is appropriate for the investigation of the aerodynamic behaviour of the DPI formulations since this is authorized by several pharmacopoeias (European Pharmacopoeia /Method Chapter 2.9.18, United States Pharmacopeia /Test Chapter <601>, Chinese Pharmacopoeia /Chapter <0951>) [51]. The plates of the impactor were dipped in a Span® 80 and cyclohexane (1:99, w/w) mixture then allowed to dry. The applied 28.3 ± 1 l/min flow rate during the *in vitro* test, which is a standard measurement parameter for ACI [51], was set by a mass flow meter (Flow Meter Model DFM 2000, Copley Scientific Ltd., Nottingham, UK) on the used vacuum pump (High-capacity Pump Model HCP5, Critical Flow Controller Model TPK, Copley Scientific Ltd., Nottingham, UK). For each sample, 10 mg of CIP was filled into the DPI capsules, this amount is one-tenth of the oral dose of CIP [42]. During the ACI test, Breezhaler® (Novartis, Basel, Switzerland) DPI device was applied with three [52] transparent, size 3 hard gelatin capsules (Coni-Snap®, Capsugel, Bornem, Belgium). For each capsule, inhalation for 4 s was applied two times. After the *in vitro* inhalation test, the Breezhaler®, the used DPI capsules, the mouthpiece, the throat, the eight plates (0–7) of the impactor, and the applied filter were washed with distilled water and the mass of CIP deposited on these parts was determined using an Ultraviolet-visible spectrophotometer (ATI-UNICAM UV/VIS Spectrophotometer, Cambridge, UK) at a wavelength of 276 nm. The calculation of the amount of API in the washed elements was possible in the knowledge of the applied volume of the flasks, the used dilution, the value of the absorbance and the slope of the calibration curve of CIP in the distilled water. So earlier we determined the linearity of the CIP calibration curve in this medium at 276 nm, it was: *y* = 0.0736x. The unit of the slope was mL/µg. Knowing the data detailed so far, it became possible to manually calculate the values which characterize the *in vitro* aerodynamic behaviour of the samples. The emitted fraction (EF) was expressed as the percentage ratio of the drug amount found in the ACI parts (from the mouthpiece to the filter) which otherwise corresponds to the emitted dose (ED) to the total mass of the drug recovered (from the DPI device to the filter) [53]. With the help of KaleidaGraph 4.0 (Synergy Software, Reading, PA, USA), the cumulative percentage less than the size range *versus* the effective cut-off diameter (ACI, 28.3 l/min flow rate [51]) was plotted on the log probability scale. Knowing the abscissa data for the 5 µm and 3 µm ordinate values, the amounts with a diameter of less than 5 µm and 3 µm can be expressed. The percentage ratios of these masses to the ED mean the FPF <5 µm and the FPF <3 µm [54]. The expression of the latter is even less common, however, it has already been published in several publications [28,55] since in the deep lung – in the sub-tracheal area – especially the particles below 3 µm are deposited [56]. The mass median aerodynamic diameter (MMAD) is defined as the diameter at which 50% of the particles of an aerosol by mass are larger and 50% are smaller [57]. This is obtained as the ordinate value for the 50% abscissa value. It should be noted that calculations are necessary to correlate it with the number of the applied DPI capsules, since the values for a dose will be obtained then. The GSD value was calculated from the equation shown in Fig. 1, where x is the aerodynamic diameter at 84.13% cumulative mass and y is the aerodynamic diameter at 15.87% cumulative mass [58].

#### 2.2.11 Statistical analyses

The statistical analyses were carried out with the application of the Social Science Statistics – available online [59]. The *t*-test calculations were made at the 0.05 significance level and with a one-tailed hypothesis. All reported data are means ± SD of three parallel investigations (*n* = 3).

## 3 Results and discussion

### 3.1 Structure of the samples

**Fig. 2:**
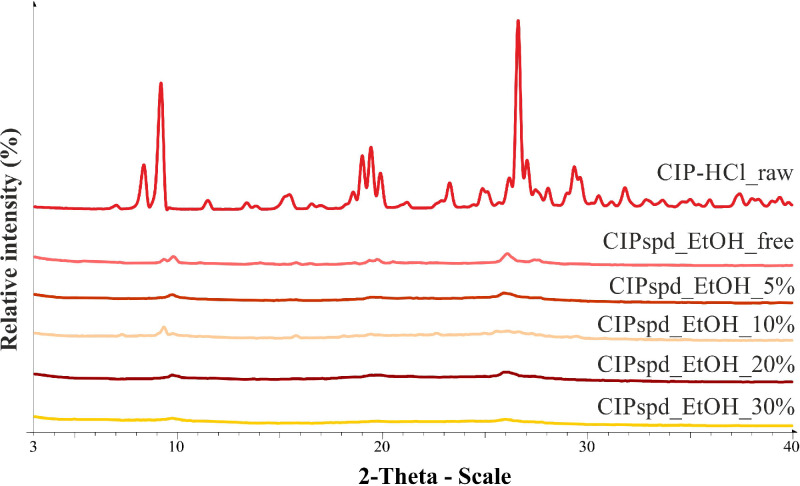
XRPD pattern of the CIP-HCl_raw and the spray-dried formulations.

The XRPD provides an opportunity to study the raw CIP (CIP-HCl_raw) and the structure of the prepared formulations (Fig. 2). The structure of the samples affects their morphology, thereby it can modify, for example, the interparticle interactions. Based on the XRPD patterns, characteristic peaks of the raw CIP are determined at 8.23, 9.25, 19.22, 26.39, and 29.16 2-Theta degree, which refers to a crystalline structure. In the case of the spray-dried samples, it is clearly visible that the characteristic peaks are present only at very low intensities, which predicts that the amorphous property predominates in these samples.

### 3.2 Morphological characterization of the samples

**Fig. 3:**
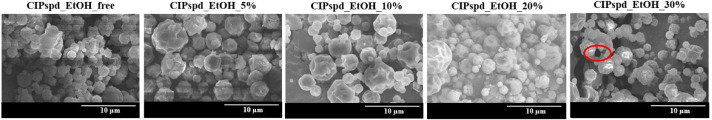
SEM recordings of the formulations.

**Fig. 4:**
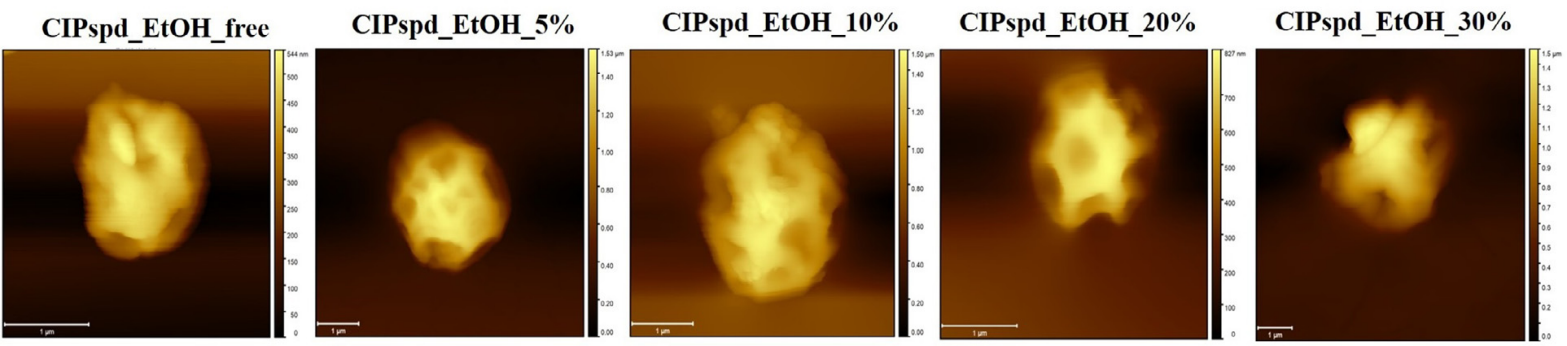
Individual particle recordings with AFM for each formulation.

However, the different EtOH concentrations of the spray-drying stock solution caused remarkable morphological differences. By increasing the EtOH concentration, the depressions in the surface of the particles increase, and cracking occurs already at 30% EtOH content – in the stock solution –. This is due to the fact that water and EtOH have different evaporation rates. At higher EtOH concentrations, higher internal pressures can cause more swollen or exploded particles [33]. Thus, the mixtures of these solvents in different proportions in the stock solution lead to the formation of different morphologies during the drying process under the same conditions. The Peclet number (Pe), which is a dimensionless value, is also linked here in an explanatory way [60]. Its calculation is based on the evaporation rate of the solvent (k, also known as convection) and the diffusion rate (D) of each dissolved molecule (Pe = *k*/8D) [61]. The diffusion coefficient may vary during spray-drying, depending on the concentration of the applied drug and the composition of the solvent, so the Pe number is not a constant value for a given material [14]. For DPIs, their exact determination is not typical in publications [60,62]. Based on observations, it can be said that if Pe<1, a compact particle with a uniform internal structure is formed. In formulations where Pe>1, the diffusion of the solute at the center of the drop is slower than the evaporation of the solvent, which leads to wavy surface particles [14]. Based on these data and SEM (Fig. 3), AFM (Fig. 4) recordings, as well as the results reported in Table 2, it can be assumed that Pe also increases with increasing EtOH concentration. Furthermore, the above-mentioned may predict different density values of the formulations.

**Table 2:** Roughness, depth and width of the dimples with respect to particle size.

	CIPspd_EtOH_free	CIPspd_EtOH_5%	CIPspd_EtOH_10%	CIPspd_EtOH_20%	CIPspd_EtOH_30%
Roughness (%)	0.85 ± 0.09	2.37 ± 0.11	2.66 ± 0.16	4.10 ± 0.18	4.46 ± 0.36
Depth of the dimples (%)	0.83 ± 0.04	2.81 ± 0.19	2.93 ± 0.21	5.73 ± 0.51	8.36 ± 0.71
Width of the dimples (%)	4.04 ± 0.17	22.56 ± 1.52	22.85 ± 1.54	25.94 ± 2.08	28.34 ± 2.32

The particle morphology from the SEM recordings (Fig. 3) was correlated with the AFM studies. AFM imaging was used to provide more details on the topography and roughness of the single particles (Fig. 4). While the surface structure of both imaging techniques correlates very well, the quantification of the morphological differences between the samples could only be evaluated via AFM analysis (Table 2). Percentages of the roughness, depth and width of the dimples for particle size were determined as described in Section 2.2.4. To the best of our knowledge, this adapted evaluation has not been applied in the literature yet. The calculation carried out allows realistic comparability between samples as it considers the ratio between the individual particle size and the roughness or the depth of the dimples. Our results showed that the roughness and the depth of the dimples increase in direct proportion to the EtOH concentration used in the stock solution. Regarding the width of the dimples, it can be seen that the presence of EtOH has already resulted in very wide dimples, which increased somewhat with higher EtOH content. Overall, increased concentration of EtOH in the stock solutions compared to the almost uniform surface of CIPspd_EtOH_free resulted in deepening/nearly equal average width dimples relative to the particle size. However, in the case of higher EtOH concentration, the standard deviation of the mentioned test values also increased, which indicates the presence of more varied shaped particles in the formulations. The explanation for these observations is that the solid shell is less and less able to follow the thermal movement of the still-drying interior using the increased EtOH concentration in the starting solution, so that the uneven surface becomes more and more pronounced.

### 3.3 Particle size distribution and Span values

The particle size distribution of the samples is shown in Table 3. For DPIs, the average particle size (D0.5) and particle size distribution are very important factors to achieve an effective aerodynamic result of the formulations [63]. Even a very small D_0.5_ deviation as well as a wider range of particle size distributions can have a remarkable effect on the therapeutic efficacy of the microcomposites [63,64]. According to the international literature, D_0.5_ values of 1–5 µm [65,66] and in some publications already 1–10 µm [67,68] are recommended for pulmonary drug administration. All formulations tested corresponded to these recommendations and the particle size distribution also ranged from 1 to 10 µm based on the D_0.1_ and D_0.9_ results. As already noted in Section 3.2, the presence of EtOH in the stock solution results in more swollen, even cracked particles, which was supported by the D_0.5_ results, which can be explained by the fact that water and EtOH have different evaporation rates [33]. Although EtOH can have a remarkably reducing effect on the surface tension of stock solutions [69], which has a positive effect on atomization [70] and can result in a smaller droplet size [71]. However, in the present case, the evaporation rate of the solvent/solvent mixtures proves to be a more notable factor in terms of particle size. After all, it can be clearly seen that the lowest value was in the case of the CIPspd_EtOH_free sample (solid, shrunken as explained in Section 3.2) (D_0.5_: 3.168 µm). Samples containing EtOH in the stock solution ranged from 3.521 to 4.317. In the case of the CIPspd_EtOH_5% sample, a slightly outstanding value can be seen in terms of D_0.5_, this phenomenon has already been observed by others when applying a small amount of organic solvent during spray-drying [33]. Then, after some decline, the D_0.5_ value increased in direct proportion to the EtOH concentration. In terms of these results and the observations reported by Ji et al. [35] and Belotti, et al. [33] it can be concluded that the use of an organic solvent (even in proportions) instead of water during spray drying does not always result in a reduction in particle size, as previously assumed [71,72]. The Span values of the samples were expressed based on Eq. (5). The lowest value was obtained for the CIPspd_EtOH_30% formulation, which shows that it is less polydisperse compared to the other samples [73].

**Table 3:** Particle size distribution of the samples.

Formulations	D_0.1_ (µm)	D_0.5_ (µm)	D_0.9_ (µm)	Span
CIPspd_EtOH_free	1.683 ± 0.08	3.168 ± 0.06	6.131 ± 0.12	1.339
CIPspd_EtOH_5%	2.177 ± 0.12	4.197 ± 0.08	7.966 ± 0.21	1.379
CIPspd_EtOH_10%	1.635 ± 0.06	3.521 ± 0.04	7.102 ± 0.07	1.553
CIPspd_EtOH_20%	1.979 ± 0.04	3.753 ± 0.09	6.846 ± 0.15	1.297
CIPspd_EtOH_30%	2.334 ± 0.13	4.317 ± 0.11	7.495 ± 0.08	1.182

### 3.4 Bulk density, tap density, Carr index and Hausner factor of the samples

For DPIs, the value of 0.3 g/cm^3^ can be considered as a limit, because experimental results prove that above this good aerodynamic results *e.g.* high respirable fraction difficult to obtain [74]. In the case of bulk density, Simon et al. [28] mention it as a goal to be achieved during development, and Zhou et al. [75] state for tapped density that the formulation is suitable for pulmonary use only below this value. In terms of results (Table 4), both bulk and tapped density values meet these reporting requirements. It is clear that CIPspd_EtOH_free has slightly higher density values than the others. Even a small increase in density can have a large effect on *in vitro* aerodynamic properties [76]. With regard to the Carr index and the Hausner ratio, it can be stated that there is no remarkable difference between the values; their flowability property should be classified into the very poor category [77]. These are not conspicuous phenomena for DPIs, and such CI and HF values have been reported in several publications [47,78,79]. It is also highly dependent on the raw active ingredient. Of course, these values can also be improved during co-spray-drying with various excipients, but here the aim was merely to study the ability of EtOH to influence the habit of the samples.

**Table 4:** Density values of the samples and expressions calculated from them.

Formulations	Bulk density (g/cm^3^)	Tapped density (g/cm^3^)	Carr index	Hausner ratio
CIPspd_EtOH_free	0.188 ± 0.02	0.294 ± 0.02	36.00	1.56
CIPspd_EtOH_5%	0.171 ± 0.01	0.269 ± 0.03	36.37	1.57
CIPspd_EtOH_10%	0.167 ± 0.03	0.255 ± 0.05	34.61	1.53
CIPspd_EtOH_20%	0.172 ± 0.02	0.270 ± 0.03	36.30	1.57
CIPspd_EtOH_30%	0.170 ± 0.01	0.264 ± 0.04	35.61	1.55

### 3.5 Residual water and EtOH content values

The residual water content of the samples was between (3.5% and 4.1%). In the case of spray-dried DPIs, for example, values ranging from 0.24% [80] to 9.02% [33] are reported in the literature. Thus, the values obtained can be considered adequate. The low water content is necessary for DPI formulations to be able to aerosolize and disperse and reach the lungs properly [81]. For samples containing EtOH in the stock solution, the residual EtOH content was below 0.4% in all cases. This is appropriate as it can be a maximum of 0.5% according to ICH Q3C(R6) guideline [82].

### 3.6 Cohesion work results

Regarding the W_c_ of the samples, we can see that the value obtained for CIPspd_EtOH_free is relatively high, which can be explained by the spherical particles with almost no depressions on the surface (see Table 5). For CIPspd_EtOH_5% and CIPspd_EtOH_10%, the W_c_ value increased, representing approximately the same value. The explanation for this can also be interpreted based on the schematic diagram, since the particles can come into contact with a larger area due to the wavy surface and the almost identical surface morphology (including the degree of the roughness, depth and width of dimples). This relationship can also be found in the publication of Lechanteur and Evrard [61]. However, for the CIPspd_EtOH_20% and CIPspd_EtOH_30% formulations, we see a remarkable decrease in W_c_, which may be explained by the fact that the SEM images show a difference in the morphology of the individual particles, as shown by the higher deviation of AFM dimples, as a result of which the connection of the particles is more difficult could result in a decrease in interparticle interactions (W_c_). Basically, the presence of dimples is described as positive in the literature for aerodynamic results [44], however, they allow greater connection for particles with the same morphology. Thus, presumably, larger dimples (wide and deep), a slightly different morphology per particle, and more favorable W_c_ values predict relatively favorable aerodynamic results for the CIPspd_EtOH_20% and CIPspd_EtOH_30% samples.

**Table 5:** Morphological properties of the samples.

	CIPspd_EtOH_free	CIPspd_EtOH_5%	CIPspd_EtOH_10%	CIPspd_EtOH_20%	CIPspd_EtOH_30%
W_c_ [mN/m]	149.68	156.64	155.40	135.72	128.34
Schematic pictures					

### 3.7 In vitro aerodynamic properties

The *in vitro* inhalation test with ACI was performed at 28.3 ± 1 l/min, which is indicated as a standard parameter in pharmacopoeias [58]. As a result of the testing, the FPF <5 µm, FPF <3 µm, MMAD, ET and GSD values (Table 6) were expressed based on the calculations detailed in Section 2.2.10. It can be clearly seen that the FPF values changed in direct proportion to the MMAD values. As a result, the FPF <5 µm, FPF <3 µm values were remarkably lower for CIPspd_EtOH_5% and CIPspd_EtOH_10% samples, approximately half of the results obtained for the CIPspd_EtOH_free formulation. Compared to these, FPF values show a substantial improvement in lung deposition results at CIPspd_EtOH_20% and CIPspd_EtOH_30%. The last one achieved the best results – FPF <5 µm (34.39%) and FPF <3 µm (15.18%). In terms of EF, the *in vitro* aerodynamic particle size distribution (APSD) testing requires this value to be between 85% and 115% [83]. In the case of the examined microcomposites, the CIPspd_EtOH_free, CIPspd_EtOH_5% and CIPspd_EtOH_30% samples met this requirement. In respect of GSD, the values above 1.2 are considered polydisperse in the literature [84]. Thus, all formulations produced have a heterogeneous particle distribution. For aerosols, GSD <2 is desirable [85], however, most therapeutic aerosols have GSD values between 2 and 3 [86]. Based on these, it can be said about the formulations reported in the publication that their GSD values, which are also between 2 and 3, are acceptable, but the most favorable value is calculated at CIPspd_EtOH_30%, which may be related to the fact that this sample also had the smallest Span value.

**Table 6:** Aerodynamic properties of the formulations.

Formulations	FPF < 5 µm(%)	FPF< 3 µm (%)	MMAD (µm)	EF (%)	GSD
CIPspd_EtOH_free	23.58 ± 0.73	9.53 ± 0.11	7.62 ± 0.13	86.26 ± 0.44	2.26 ± 0.04
CIPspd_EtOH_5%	11.96 ± 0.16	4.85 ± 0.23	12.03 ± 0.08	89.57 ± 0.31	2.72 ± 0.11
CIPspd_EtOH_10%	13.05 ± 0.25	5.85 ± 0.17	10.18 ± 0.16	78.13 ± 0.83	2.37 ± 0.06
CIPspd_EtOH_20%	28.51 ± 0.43	12.27 ± 0.33	5.83 ± 0.03	75.80 ± 0.65	2.06 ± 0.01
CIPspd_EtOH_30%	34.39 ± 0.54	15.18 ± 0.42	5.21 ± 0.11	87.12 ± 0.39	2.01 ± 0.03

**Fig. 5:**
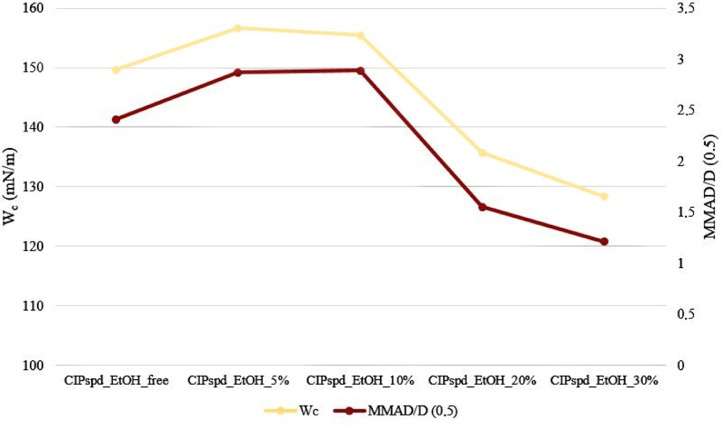
Correlation between W_c_ and MMAD/D_0.5_.

An actual explanation of the aerodynamic results is given in Fig. 5. The study of the habit of the particles has already detailed how the different EtOH concentrations modify the D_0.5_ values and what morphological changes they result in, the latter influencing the W_c_ results. Fig. 5. highlights the correlation between the trends of W_c_ and MMAD/D_0.5_ values. Thus, it was confirmed how many times the MMAD value of the D_0.5_ result per formulation became a function of the W_c_ values. As a result, it is understandable that the D_0.5_ values did not correlate directly with the FPF results, however, for example, the relatively low D_0.5_ value (3.168 µm) and high W_c_ value (149.68 mN/m) of CIPspd_EtOH_free resulted in an MMAD of 7.62 µm (2.41 time the D_0.5_ value), even in the case of CIPspd_EtOH_5% a higher D_0.5_ value (4.197 µm) and also a high W_c_ value (156.64 mN/m) were shown in the higher MMAD result (12.03 µm). Furthermore, in the case of CIPspd_EtOH_30%, we can see that although it has the highest D_0.5_ value (4.317 µm) of the examined formulations, due to the substantially lower W_c_ value (128.34 mN/m), the MMAD result was the most favorable (only 1.21 time the D_0.5_ value). To the best of our knowledge, the above-mentioned relationship between W_c_ and MMAD/D_0.5_ has not been reported in this form in the international literature. From the MMAD value, the FPF values followed in direct proportion.

## 4 Conclusion

A comprehensive study was introduced from the field of DPI formulations containing various concentrations of EtOH using CIP as an antibiotic agent. The results clearly show that mainly formally different particles were produced during the preparation process. By using water for sample preparation and subsequently applying the spray-drying process, (CIPspd_EtOH_free), small shrunk particles were formed that showed a slightly higher density compared to the samples prepared with EtOH. From the morphological point of view, the particles were spherical without substantial surface depressions, resulting in a high W_c_ value. As a result, an FPF <5 µm of 23.58% was obtained. Using low EtOH concentrations (5%, 10%), higher D_0.5_ (the particles are already swollen) and W_c_ (due to their morphological properties) values were achieved, which together resulted in a remarkable relapse in the aerodynamic results (MMAD, FPF). The formulations produced with 20% and 30% EtOH resulted in high D_0.5_ values, however, due to the varied morphology, substantially lower W_c_ values could be measured, not favouring the aggregation of the particles, so the MMAD results and consequently the FPF values were already more favourable. For CIP, the use of 30% EtOH in stock solution resulted in the most favourable *in vitro* aerodynamic results, matching the requirements and guideline values for DPIs.

This study was not intended to illustrate the development of a final DPI formulation (therefore, we did not use solid excipients for co-spray drying), but highlighted the influence of the composition of the solvent mixture on the properties of carrier-free formulations prepared via spray-drying. Therefore, differences in FPF values were obtained *in vitro* aerodynamic tests, which draws attention to the need to prioritize *e.g.* for the effect of the applied solvent concentrations during the development of DPIs. The results reported are supported for CIP, but the effect of solvents/solvent mixtures on DPI formulations requires prior investigation for each active ingredient. Furthermore, the new approaches detailed in the present work, *i.e.* monitoring the MMAD/D_0.5_ ratio and correlating it with W_c_, moreover, the expression of the roughness, depth and width of the dimples for particle size values may be proposed for all carrier-free DPIs, which may point to new correlations during development.

## Conflicts of interest

The authors report no conflicts of interest. The authors alone are responsible for the content and writing of this article.

## References

[1] Weber S., Zimmer A., Pardeike J. (2014). Solid lipid nanoparticles (SLN) and nanostructured lipid carriers (NLC) for pulmonary application: a review of the state of the art. Eur J Pharm Biopharm.

[2] Paranjpe M., Müller-Goymann C. (2014). Nanoparticle-mediated pulmonary drug delivery: a review. IJMS.

[3] D. Ganderton, M.J. Main, F.G. Morgan Compositions for treating Parkinson's disease. WO2009056851A1[P]. 2009.

[4] Momin M.N., Hedayati A., Nokhodchi A. (2011). Investigation into alternative sugars as potential carriers for dry powder formulation of budesonide. BioImpacts.

[5] Chen L., Okuda T., Lu X.Y., Chan H.K. (2016). Amorphous powders for inhalation drug delivery. Adv Drug Deliv Rev.

[6] Jones B., Ecenarro Probst S. (2017). An overview of dry powder inhalation for systemic drug delivery. ONDrugDelivery Mag.

[7] Borghardt J.M., Kloft C., Sharma A. (2018). Inhaled therapy in respiratory disease: the complex interplay of pulmonary kinetic processes. Can Respir J.

[8] Sibum I., Hagedoorn P., de Boer A.H., Frijlink H.W., Grasmeijer F. (2018). Challenges for pulmonary delivery of high powder doses. Int J Pharm.

[9] Mehta P. (2016). Dry powder inhalers: a focus on advancements in novel drug delivery systems. J Drug Deliv.

[10] Sanjeevani Shekhar D., Alisha Swizal V. (2019). Recent updates on dry powder for inhalation for pulmonary drug delivery systems. IJRPS.

[11] Demoly P., Hagedoorn P., de Boer A.H., Frijlink H.W. (2014). The clinical relevance of dry powder inhaler performance for drug delivery. Respir Med.

[12] Abadelah M., Chrystyn H., Bagherisadeghi G., Abdalla G., Larhrib H. (2018). Study of the rmitted dose after two separate inhalations at different inhalation flow rates and volumes and an assessment of aerodynamic characteristics of indacaterol Onbrez Breezhaler® 150 and 300µg. AAPS Pharm Sci Tech.

[13] Liang W., Chan A.Y.L., Chow M.Y.T., Lo F.F.K., Qiu Y., Kwok P.C.L. (2018). Spray freeze drying of small nucleic acids as inhaled powder for pulmonary delivery. Asian J Pharm Sci.

[14] Chvatal A., Benke E., Szabó-Révész P., Ambrus R. (2016). New strategies of DPI formulations. Gyógyszerészet/Pharmacy.

[15] Depreter F., Pilcer G., Amighi K. (2013). Inhaled proteins: challenges and perspectives. Int J Pharm.

[16] Kozáková J., Altay A., Ždímal V., Mašková L., Sonvico F., Quarta E. (2019). Dry powder inhaler of colistimethate sodium for lung infections in cystic fibrosis: optimization of powder construction. Drug Dev Ind Pharm.

[17] Yang X.F., Xu Y., Qu D.S., Li H.Y. (2015). The influence of amino acids on aztreonam spray-dried powders for inhalation. Asian J Pharm Sci.

[18] Trotta V., Lee W.H., Loo C.Y., Young P.M., Traini D., Scalia S. (2016). Co-spray dried resveratrol and budesonide inhalation formulation for reducing inflammation and oxidative stress in rat alveolar macrophages. Eur J Pharm Sci.

[19] Lu W., Rades T., Rantanen J., Yang M. (2019). Inhalable co-amorphous budesonide-arginine dry powders prepared by spray drying. Int J Pharm.

[20] Pomázi A., Buttini F., Ambrus R., Colombo P., Szabó-Révész P. (2013). Effect of polymers for aerolization properties of mannitol-based microcomposites containing meloxicam. Eur Polym J.

[21] Liang Z., Ni R., Zhou J., Mao S. (2015). Recent advances in controlled pulmonary drug delivery. Drug Discov Today.

[22] Yang Y., Cheow W.S., Hadinoto K. (2012). Dry powder inhaler formulation of lipid–polymer hybrid nanoparticles via electrostatically-driven nanoparticle assembly onto microscale carrier particles. Int J Pharm.

[23] Raula J., Rahikkala A., Halkola T., Pessi J., Peltonen L., Hirvonen J. (2013). Coated particle assemblies for the concomitant pulmonary administration of budesonide and salbutamol sulphate. Int J Pharm.

[24] Vehring R. (2008). Pharmaceutical particle engineering via spray drying. Pharm Res.

[25] Li L., Sun S., Parumasivam T., Denman J.A., Gengenbach T., Tang P. (2016). L-Leucine as an excipient against moisture on *in vitro* aerosolization performances of highly hygroscopic spray-dried powders. Eur J Pharm Biopharm.

[26] Minne A., Boireau H., Horta M.J., Vanbever R. (2008). Optimization of the aerosolization properties of an inhalation dry powder based on selection of excipients. Eur J Pharm Biopharm.

[27] Prota L., Santoro A., Bifulco M., Aquino R.P., Mencherini T., Russo P. (2011). Leucine enhances aerosol performance of Naringin dry powder and its activity on cystic fibrosis airway epithelial cells. Int J Pharm.

[28] Simon A., Amaro M.I., Cabral L.M., Healy A.M., de Sousa V.P. (2016). Development of a novel dry powder inhalation formulation for the delivery of rivastigmine hydrogen tartrate. Int J Pharm.

[29] Zhu B., Haghi M., Nguyen A., Goud M., Yeung S., Young P.M. (2015). Delivery of theophylline as dry powder for inhalation. Asian J Pharm Sci.

[30] Peng T., Zhang X., Huang Y., Zhao Z., Liao Q., Xu J. (2017). Nanoporous mannitol carrier prepared by non-organic solvent spray drying technique to enhance the aerosolization performance for dry powder inhalation. Sci Rep.

[31] Azari F., Ghanbarzadeh S., Safdari R., Yaqoubi S., Adibkia K., Hamishehkar H. (2020). Development of a carrier free dry powder inhalation formulation of Ketotifen for pulmonary drug delivery. Drug Res (Stuttg).

[32] Ungaro F., Giovino C., Coletta C., Sorrentino R., Miro A., Quaglia F. (2010). Engineering gas-foamed large porous particles for efficient local delivery of macromolecules to the lung. Eur J Pharm Sci.

[33] Belotti S., Rossi A., Colombo P., Bettini R., Rekkas D., Politis S. (2015). Spray-dried amikacin sulphate powder for inhalation in cystic fibrosis patients: the role of ethanol in particle formation. Eur J Pharm Biopharm.

[34] Gilani K., Najafabadi A.R., Barghi M., Rafiee-Tehrani M. (2005). The effect of water to ethanol feed ratio on physical properties and aerosolization behavior of spray dried cromolyn sodium particles. J Pharm Sci.

[35] Ji S., Thulstrup P.W., Mu H., Hansen S.H., van de Weert M., Rantanen J. (2016). Effect of ethanol as a co-solvent on the aerosol performance and stability of spray-dried lysozyme. Int J Pharm.

[36] Rabbani N.R., Seville P.C. (2005). The influence of formulation components on the aerosolisation properties of spray-dried powders. J Control Release.

[37] Boraey M.A., Hoe S., Sharif H., Miller D.P., Lechuga-Ballesteros D., Vehring R. (2013). Improvement of the dispersibility of spray-dried budesonide powders using leucine in an ethanol–water cosolvent system. Powder Technol.

[38] Parlati C., Colombo P., Buttini F., Young P.M., Adi H., Ammit A.J. (2009). Pulmonary spray dried powders of tobramycin containing sodium stearate to improve aerosolization efficiency. Pharm Res.

[39] Nguyen T.T., Yi E.J., Hwang K.M., Cho C.H., Park C.W., Kim J.Y. (2019). Formulation and evaluation of carrier-free dry powder inhaler containing sildenafil. Drug Deliv Transl Res.

[40] Son Y.J., Worth Longest P., Hindle M. (2013). Aerosolization characteristics of dry powder inhaler formulations for the excipient enhanced growth (EEG) application: effect of spray drying process conditions on aerosol performance. Int J Pharm.

[41] Karimi K., Pallagi E., Szabó-Révész P., Csóka I., Ambrus R. (2016). Development of a microparticle-based dry powder inhalation formulation of ciprofloxacin hydrochloride applying the quality by design approach. Drug Des Dev Ther.

[42] Benke E., Farkas Á., Balásházy I., Szabó-Révész P., Ambrus R. (2019). Stability test of novel combined formulated dry powder inhalation system containing antibiotic: physical characterization and *in vitro* – *in silico* lung deposition results. Drug Dev Ind Pharm.

[43] Yang J.J., Liu C.Y., Quan L.H., Liao Y.H. (2012). Preparation and *in vitro* aerosol performance of spray-dried Shuang-Huang-Lian corrugated particles in carrier-based dry powder inhalers. AAPS PharmSciTech.

[44] Adi H., Traini D., Chan H.K., Young P.M. (2008). The influence of drug morphology on aerosolisation efficiency of dry powder inhaler formulations. J Pharm Sci.

[45] Zhu B., Padroni M., Colombo G., Phillips G., Crapper J., Young P.M. (2016). The development of a single-use, capsule-free multi-breath tobramycin dry powder inhaler for the treatment of cystic fibrosis. Int J Pharm.

[46] Ungaro F., De Rosa G., Miro A., Quaglia F., La Rotonda M.I (2006). Cyclodextrins in the production of large porous particles: development of dry powders for the sustained release of insulin to the lungs. Eur J Pharm Sci.

[47] Mehanna M.M., Mohyeldin S.M., Elgindy N.A. (2019). Rifampicin-carbohydrate spray-dried nanocomposite: a futuristic multiparticulate platform for pulmonary delivery. IJN.

[48] Farkas B., Révész P. (2007).

[49] Schuster J.M., Schvezov C.E., Rosenberger M.R. (2015). Analysis of the results of surface free energy measurement of Ti6Al4V by different methods. Proc Mater Sci.

[50] Tüske Z. (2005). https://doktori.bibl.u-szeged.hu/id/eprint/242.

[51] Inhaler Testing Brochure 2020. Copley Scientific 2020. Available from: https://www.copleyscientific.com/downloads/inhaler-testing-brochure-2020/.

[52] Li X., Vogt F.G., Hayes D., Mansour H.M. (2014). Physicochemical characterization and aerosol dispersion performance of organic solution advanced spray-dried microparticulate/nanoparticulate antibiotic dry powders of tobramycin and azithromycin for pulmonary inhalation aerosol delivery. Eur J Pharm Sci.

[53] Benke E., Farkas Á., Szabó-Révész P., Ambrus R. (2020). Development of an innovative, carrier-based dry powder inhalation formulation containing spray-dried meloxicam potassium to improve the *in vitro* and in silico aerodynamic properties. Pharmaceutics.

[54] Cunha L., Rodrigues S., Rosa da Costa A., Faleiro M., Buttini F., Grenha A. (2018). Inhalable fucoidan microparticles combining two antitubercular drugs with potential application in pulmonary tuberculosis therapy. Polymers.

[55] Ceschan N.E., Bucalá V., Mateos M.V., Smyth H.D.C., Ramírez-Rigo M.V. (2018). Carrier free indomethacin microparticles for dry powder inhalation. Int J Pharm.

[56] Hoffelder T., Wellek S. (2017). Equivalence testing with particle size distribution data: methods and applications in the development of inhalative drugs. Stat Biopharm Res.

[57] Laube B.L., Janssens H.M., de Jongh F.H.C., Devadason S.G., Dhand R., Diot P. (2011). What the pulmonary specialist should know about the new inhalation therapies. Eur Respir J.

[58] Yoshida H., Kuwana A., Shibata H., Izutsu K., Goda Y. (2017). Comparison of aerodynamic particle size distribution between a next generation impactor and a cascade impactor at a range of flow rates. AAPS PharmSciTech.

[59] Social science statistic online: 2020. Available from: https://www.socscistatistics.com/tests/studentttest/default2.aspx.

[60] Šimková K., Joost B., Imanidis G. (2020). Production of fast-dissolving low-density powders for improved lung deposition by spray drying of a nanosuspension. Eur J Pharm Biopharm.

[61] Lechanteur A., Evrard B. (2020). Influence of Composition and spray-drying process parameters on carrier-free DPI properties and behaviors in the lung: a review. Pharmaceutics.

[62] Berkenfeld K., McConville J.T., Lamprecht A. (2020). Inhalable formulations of rifampicin by spray drying of supersaturated aqueous solutions. Eur J Pharm Biopharm.

[63] Peng T., Lin S., Niu B., Wang X., Huang Y., Zhang X. (2016). Influence of physical properties of carrier on the performance of dry powder inhalers. Acta Pharm Sin B.

[64] Saiful Hassan M., Lau R. (2010). Effect of particle formulation on dry powder inhalation efficiency. CPD.

[65] Gajjar P., Styliari I.D., Nguyen T.T.H., Carr J., Chen X., Elliott J.A. (2020). 3D characterisation of dry powder inhaler formulations: developing X-ray micro computed tomography approaches. Eur J Pharm Biopharm.

[66] D.R. Elmaleh Dry-powder inhalation device. USA, 10238820[P].2019-3-26.

[67] Chvatal A., Farkas Á., Balásházy I., Szabó-Révész P., Ambrus R. (2017). Aerodynamic properties and in silico deposition of meloxicam potassium incorporated in a carrier-free DPI pulmonary system. Int J Pharm.

[68] Chvatal A., Ambrus R., Party P., Katona G., Jójárt-Laczkovich O., Szabó-Révész P. (2019). Formulation and comparison of spray dried non-porous and large porous particles containing meloxicam for pulmonary drug delivery. Int J Pharm.

[69] Vazquez G., Alvarez E., Navaza J.M. (1995). Surface tension of alcohol water + water from 20 to 50 °C. J Chem Eng Data.

[70] Santos D., Maurício A.C., Sencadas V., Santos J.D., Fernandes M.H., Gomes P.S., Pignatello R, Musumeci T (2018). Biomaterials - physics and chemistry.

[71] Arpagaus C. (2019). PLA/PLGA nanoparticles prepared by nano spray drying. J Pharm Investig.

[72] Patel B.B., Patel J.K., Chakraborty S., Shukla D. (2015). Revealing facts behind spray dried solid dispersion technology used for solubility enhancement. Saudi Pharm J.

[73] Kaialy W., Alhalaweh A., Velaga S.P., Nokhodchi A. (2012). Influence of lactose carrier particle size on the aerosol performance of budesonide from a dry powder inhaler. Powder Technol.

[74] Suzuki É.Y., Amaro M.I., de Almeida G.S., Cabral L.M., Healy A.M., de Sousa V.P. (2018). Development of a new formulation of roflumilast for pulmonary drug delivery to treat inflammatory lung conditions. Int J Pharm.

[75] Zhou (Tony) Q., Tang P., Leung S.S.Y., Chan J.G.Y., Chan H.K (2014). Emerging inhalation aerosol devices and strategies: where are we headed?. Adv Drug Deliv Rev.

[76] Kulvanich P., Sinsuebpol C., Chatchawalsaisin J. (2013). Preparation and *in vivo* absorption evaluation of spray dried powders containing salmon calcitonin loaded chitosan nanoparticles for pulmonary delivery. DDDT.

[77] Goyal A., Sharma V., Sihag M.K., Tomar S.K., Arora S., Sabikhi L. (2015). Development and physico-chemical characterization of microencapsulated flaxseed oil powder: a functional ingredient for omega-3 fortification. Powder Technol.

[78] Chougule M., Padhi B., Misra A. (2008). Development of spray dried liposomal dry powder inhaler of dapsone. AAPS PharmSciTech.

[79] Salem A., ME A., Eid K., Sharaf M.A. (2011). Nanosized rods agglomerates as a new approach for formulation of a dry powder inhaler. IJN.

[80] Li X., Vogt F.G., Hayes D., Mansour H.M. (2014). Design, characterization, and aerosol dispersion performance modeling of advanced co-spray dried antibiotics with mannitol as respirable microparticles/nanoparticles for targeted pulmonary delivery as dry powder inhalers. J Pharm Sci.

[81] Meenach S.A., Anderson K.W., Zach Hilt J., McGarry R.C., Mansour H.M (2013). Characterization and aerosol dispersion performance of advanced spray-dried chemotherapeutic PEGylated phospholipid particles for dry powder inhalation delivery in lung cancer. Eur J Pharm Sci.

[82] European Medicines Agency 2018. ICH Q3C (R6) Residual solvents. Availabile from:https://www.ema.europa.eu/en/ich-q3c-r6-residual-solvents.

[83] Lewis D., Rouse T., Singh D., Edge S. (2017). https://www.americanpharmaceuticalreview.com/Featured-Articles/337338-Defining-the-Dose-for-Dry-Powder-Inhalers-The-Challenge-of-Correlating-In-Vitro-Dose-Delivery-Results-with-Clinical-Efficacy/.

[84] Lavorini F., Pedersen S., Usmani O.S., Barnes P.J., Corbetta L., Corrigan C.J. (2017). Dilemmas, confusion, and misconceptions related to small airways directed therapy. Chest.

[85] Ibrahim M., Verma R., Garcia-Contreras L. (2015). Inhalation drug delivery devices: technology update. Med Dev (Auckl NZ).

[86] Pleasants R.A., Hess D.R. (2018). Aerosol delivery devices for obstructive lung diseases. Respir Care.

